# A rare case of metastatic solitary fibrous tumor of the pancreas manifesting as a cystic neoplasm: a case report

**DOI:** 10.1186/s40792-019-0699-1

**Published:** 2019-09-13

**Authors:** Hironori Yamashita, Yasuhiro Fujino, Tadayuki Ohara, Keitaro Kakinoki, Takemi Sugimoto, Kazuyoshi Kajimoto, Masahiro Tominaga

**Affiliations:** 1 Department of Gastroenterological Surgery, Hyogo Cancer Center, 13-70 Kitaoji-Cho, Akashi-Shi, Hyogo 673-8558 Japan; 2 Department of Pathology, Hyogo Cancer Center, 13-70 Kitaoji-Cho, Akashi-Shi, Hyogo 673-8558 Japan

**Keywords:** Solitary fibrous tumor, Extrathoracic, Pancreas, Metastasis, Malignant, Cystic tumor, Hemangiopericytoma

## Abstract

**Background:**

Solitary fibrous tumor (SFT) is a rare mesenchymal tumor that typically arises from the pleura. Although it may appear in other organs, it rarely develops in the pancreas. We report herein a rare case of metastatic SFT of the pancreas originating from an intracranial tumor and subsequently identified as a cystic neoplasm of the pancreas.

**Case presentation:**

A 58-year-old woman with a past medical history of brain tumor visited the hospital for further investigation of a cystic tumor in the pancreas tail. Abdominal imaging showed a heterogeneously enhancing mass that was initially suspected as a neuroendocrine neoplasm, solid pseudopapillary neoplasm, or mucinous cystic neoplasm of the pancreas. Distal pancreatectomy was performed without any intraoperative and postoperative complications. Pathological findings confirmed a diagnosis of malignant SFT of the pancreas with hyperproliferative potential. A histopathological review of her brain tumor revealed that the pancreatic tumor was derived from her brain lesion. The patient developed recurrent brain disease 4 years after the pancreatectomy, but no recurrence has been observed in the abdominal cavity.

**Conclusions:**

SFT should be considered in the differential diagnosis of untypical hypervascular pancreatic mass, particularly in patients with a history of an intrathoracic or intracranial mesenchymal tumor. Immunohistochemical analysis is crucial in detecting this tumor entity. Hyperproliferative status indicates a malignant disease and requires careful postoperative observation.

## Background

Solitary fibrous tumor (SFT) is a rare neoplasm arising from mesenchymal cells, typically in the pleura. SFTs mostly develop in the thoracic cavity, although they can also occasionally occur in extrathoracic sites. Meanwhile, SFT of the pancreas is extremely rare, with only 21 cases reported in the English literature to date [1–21]. Majority of these pancreatic SFTs originate from the pancreas itself and have no malignant features.

Herein, we present a rare case of metastatic SFT of the pancreas that originated from a central nervous system tumor and was subsequently identified as a cystic pancreatic neoplasm.

## Case presentation

A 58-year-old woman was referred to our center for further investigation of a cystic lesion in the pancreas. She has a history of repeated resection for brain tumor diagnosed as meningioma in another hospital. The first brain surgery was performed at 31 years of age, and she underwent surgical excision of the recurrent tumor 16 and 26 years after the initial resection. During admission in a community hospital for the treatment of a femoral bone fracture, computed tomography (CT) incidentally detected a 5.5-cm cystic tumor in the pancreas tail.

The laboratory data on admission in our hospital indicated a slightly elevated level of serum lipase (56 IU/L; normal range, 13–55 IU/L) and gamma-glutamyl transpeptidase (47 IU/L; normal range, 9–32 IU/L). In contrast, the tumor marker levels of carcinoembryonic antigen, carbohydrate antigen 19-9, and DUPAN-2 were within the normal range.

**Fig. 1:**
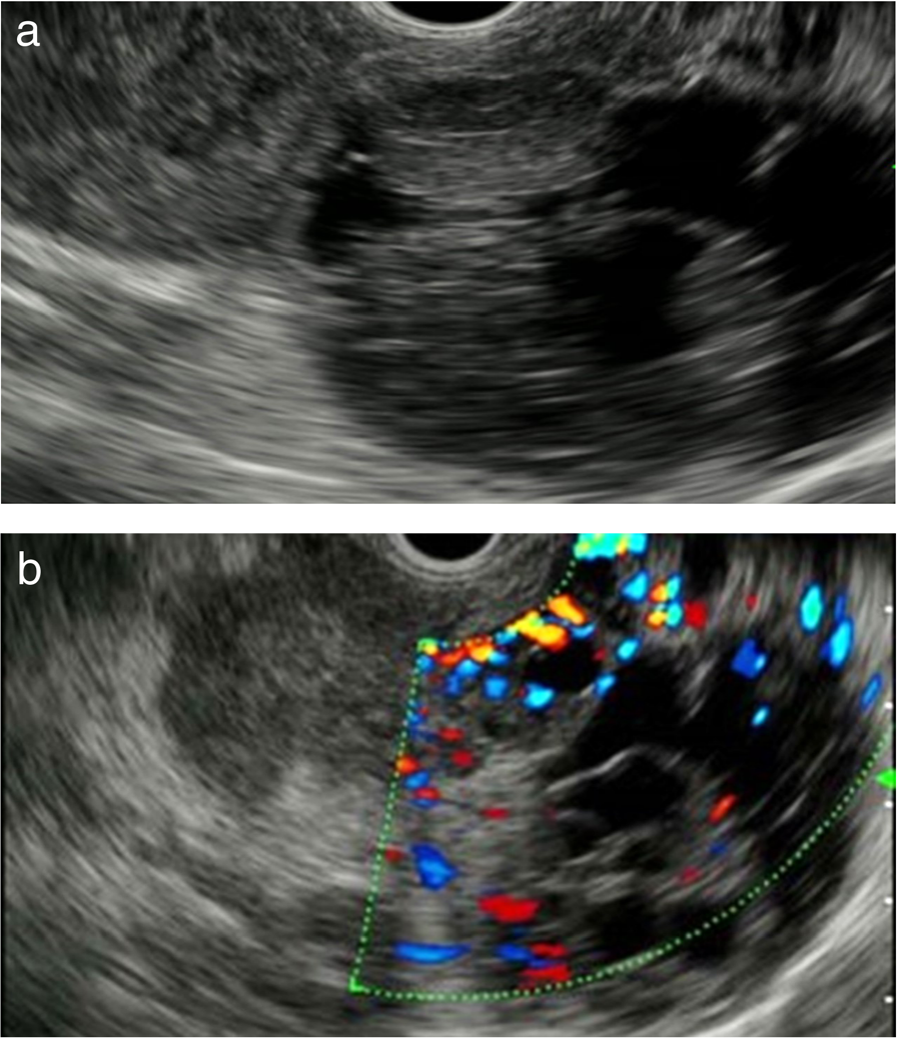
Endoscopic ultrasonography (EUS) findings. **a** EUS revealed a well-demarcated cystic mass with solid components in the pancreas tail. **b** Color Doppler EUS revealed the tumor was hypervascular, particularly on the surface

**Fig. 2:**
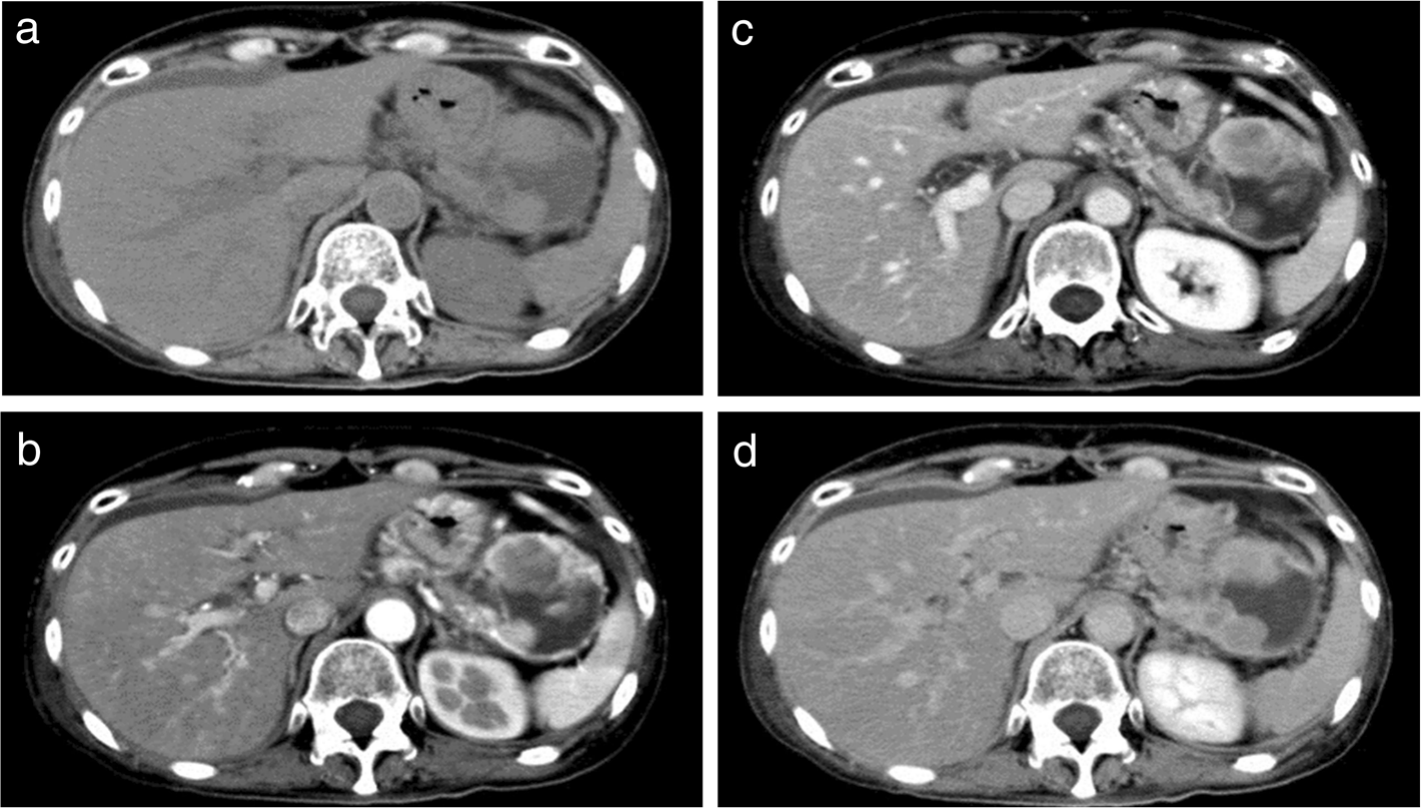
Dynamic contrast-enhanced computed tomography (CT) findings. **a** Plain CT scan demonstrated a 5.5-cm cystic mass with solid components inside in the pancreas tail. **b** Dynamic CT arterial phase scan demonstrated enhancement in the tumor rim and in the edge of the solid components with non-enhanced interior. **c** Solid components were gradually enhanced from the outside in the portal phase. **d** The tumor rim and the solid components had isodensity to the surrounding pancreatic parenchyma in the delayed phase

**Fig. 3:**
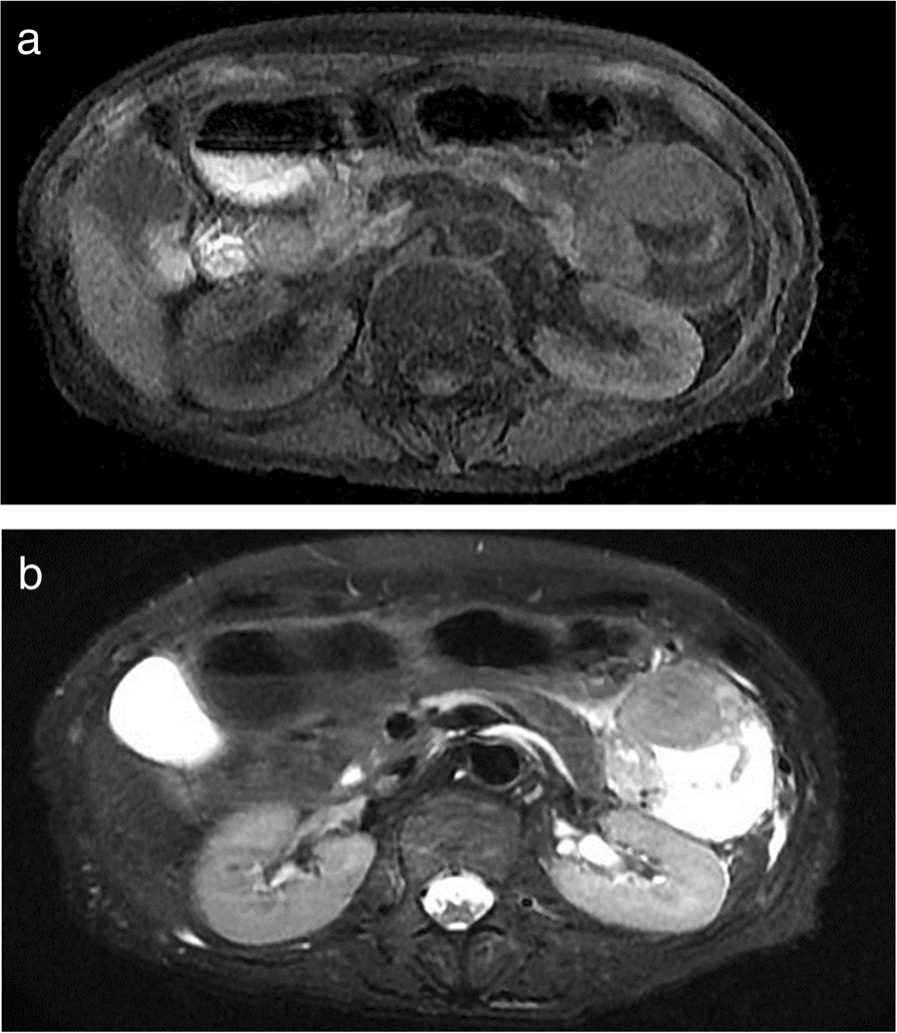
Magnetic resonance imaging (MRI) findings. **a** Solid components were hypointense to the pancreatic parenchyma on T1-weighted imaging. **b** On T2-weighted imaging, solid components were hyperintense. A large non-enhancing portion on the CT scan showed bright signal intensity, indicating cystic change or necrosis

Endoscopic ultrasonography (EUS) revealed a circumscribed well-encapsulated cystic mass in the pancreas tail with protruding vascularity-rich components inside. A hypervascular area that appeared like collateral vessels was also observed on the surface of the tumor (Fig. 1a, b). Dynamic contrast-enhanced CT showed a heterogeneously enhancing mass beside the splenic hilum with a large non-enhancing portion inside. From the arterial to portal phase, strong enhancement was observed both in the rim and the edge of the protruding solid components with the hypoattenuating area inside the solid lesions. During the portal to delay phase, all of these were gradually isoattenuated compared to the surrounding pancreatic parenchyma except for the non-enhancing portion (Fig. 2a–d). Magnetic resonance imaging (MRI) confirmed low signal intensity in the solid components on T1-weighted imaging and slightly higher signal intensity on T2-weighted imaging compared with the pancreas parenchyma. Additionally, the large non-enhancing portion on CT appeared as a bright signal on T2-weighted imaging, indicating cystic or necrotic change (Fig. 3a, b).

Based on these findings, we considered pancreatic neuroendocrine neoplasm, solid pseudopapillary neoplasm, and hemangioma in the differential diagnosis. Although its morphological structure is untypical, mucinous cystic neoplasm was also considered given the patient’s sex and tumor location. Invasive carcinoma of the pancreas was excluded from the differential diagnosis. EUS-guided fine-needle aspiration biopsy (EUS-FNA) was not performed considering the risk of cystic puncture and bleeding. We performed distal pancreatectomy with regional lymph node dissection for this disease. The pancreatic parenchyma was resected above the left edge of the superior mesenteric artery. The postoperative course was uneventful, and the patient was discharged on the 15th postoperative day.

**Fig. 4:**
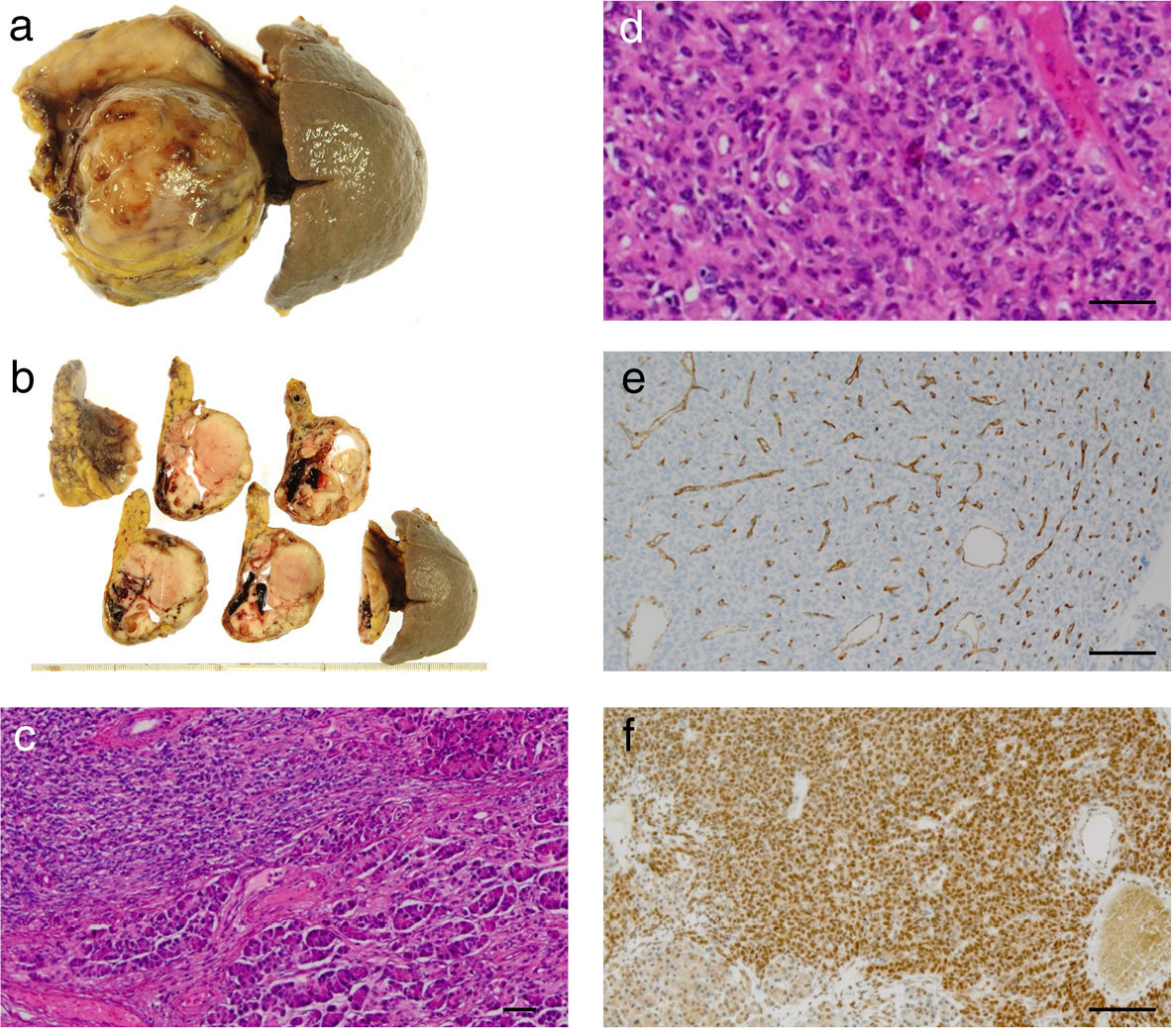
Macroscopic and microscopic findings of the resected specimen of the pancreas. **a** The resected specimen showed a hemispheric mass protruding from the pancreas tail and measuring 5.6 × 5.4 cm in diameter. **b** Solid components occupied most of this cystic mass. **c**, **d** Findings of the H&E staining. Oval and spindle-shaped cells proliferated bluntly with a richly vascular stroma, resembling the structure of hemangiopericytoma. Scale bars, 50 μm. **e** Immunohistochemical staining was irregularly positive for CD34, a mesenchymal marker. Scale bar, 100 μm. **f** Nuclear expression of STAT6 was diffusely positive in the immunohistochemical staining. Scale bar, 100 μm

The resected specimen revealed a well-demarcated hemispheric cystic mass projecting from the pancreas tail and measuring 5.6 × 5.4 cm in diameter. The solid components occupied most of the lesion (Fig. 4a, b). The pancreatic stump was free of tumor with a margin of approximately 3 cm including the width of the stapler closure. Histopathological examination confirmed that oval and spindle-shaped cells proliferated bluntly with a richly vascular stroma, and they were configured to be a hemangiopericytoma-like structure (Fig. 4c, d). Immunohistochemically, the specimen stained positively for CD34, a mesenchymal marker (Fig. 4e), but negatively for cytokeratin AE1/3, an epithelium marker (data not shown). Furthermore, CD99, Bcl-2, and STAT6 were diffusely positive (Fig. 4f), whereas beta-catenin, chromogranin, and synaptophysin were all negative (data not shown). Therefore, we diagnosed this tumor as SFT of the pancreas. Further, the tumor showed an increased mitotic rate (ten mitoses per ten high-power fields), indicating its malignant potential.

**Fig. 5:**
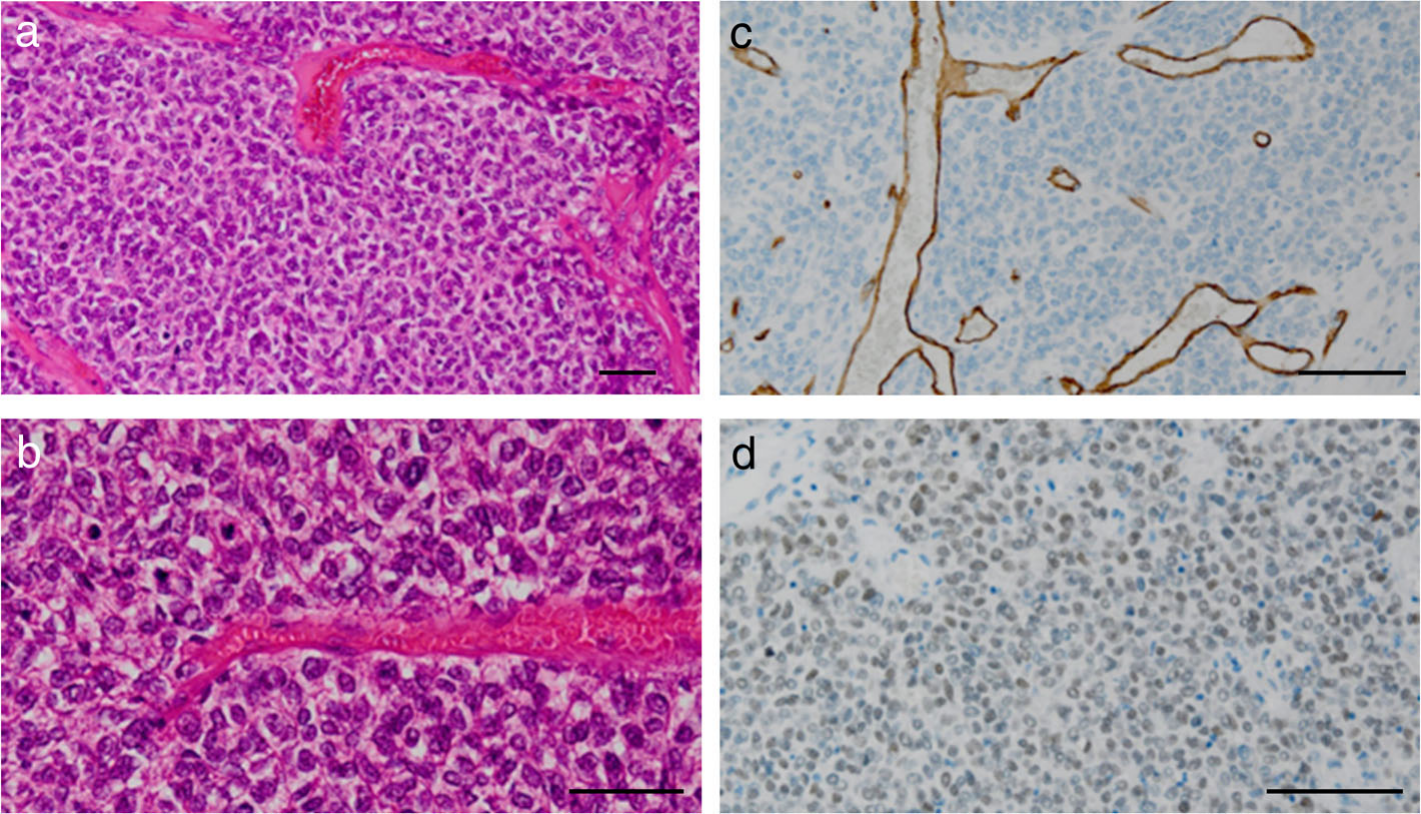
Histopathological review of the past brain tumor. **a**, **b** Findings of the H&E staining. Oval and spindle-shaped cells proliferated similarly with a richly vascular stroma. Scale bars, 50 μm. Immunohistochemical staining (**c** CD34, **d** STAT6) showed the same results as that in the pancreatic lesion. Scale bars, 100 μm

Considering the similarity of meningioma, which was the patient’s past disease, to intracranial SFT, we conducted a histopathological review of her brain tumor. We found that it had the same characteristics as the pancreatic tumor (Fig. 5) and thus determined that the SFT of the pancreas was derived from the central nervous system tumor.

She underwent no adjuvant therapy, and no recurrence has been observed in the abdominal cavity. However, she developed meningeal dissemination 4 years after the pancreatectomy and is currently undergoing treatment.

## Discussion

SFT is a rare neoplasm arising from mesenchymal cells and was first reported in 1931 by Klemperer and Rabin [22]. It is typically observed in the thoracic cavity, but an increasing number of extrathoracic lesions such as in the central nervous system [23], orbit [24], sinonasal tract [25], thyroid gland [26], liver [27], and kidney [28] have been recently reported. SFTs can develop in any organs containing mesenchymal tissues, including the pancreas [2].

Pancreatic mesenchymal tumors include leiomyosarcoma, peripheral nerve tumors, fibrous histiocytic tumors, and vascular tumors, with leiomyosarcomas being the most common [1]. Meanwhile, pancreatic SFTs are exceedingly rare, and only 21 cases have been reported in the English literature to date (Table 1). Of these, 10 were men and 12 were women, including the present case, and the median patient age was 55 years (range, 1–82 years). The primary symptom was abdominal pain or discomfort, while 50% of the patients were asymptomatic and incidentally diagnosed. Some patients presented with obstructive jaundice, but no specific symptoms could be observed. The distribution of SFT location was almost equal (pancreas head [11 cases] vs body to tail [11 cases]).

**Table 1:** Cases of primary and secondary SFT of the pancreas reported in the English literature

	Author	Year	Age, sex	Symptoms	Site	Size (cm)	Preoperative biopsy (diagnosis)	Preoperative diagnosis	Operation	Primary site	Malignant findings^a^	Postoperative course
1	Luttges et al. [1]	1999	50, F	Asymptomatic	Pb	5.5	Open biopsy (mesenchymal origin)	NEN	DP	Panc	None	20 months, no recurrence
2	Miyamoto et al. [2]	2007	41, F	Abdominal pain	Pbh	2.0	CT-guided FNA (insufficient material)	NEN	Laparoscopic EN	Panc	None	7 months, no recurrence
3	Srinivasan et al. [3]	2008	78, F	Back pain, fatigue, weight loss	Pb	5.0	EUS-FNA (mesenchymal tumor)	Mesenchymal tumor	DP	Panc	None	7 months, no recurrence
4	Kwon et al. [4]	2008	54, M	Asymptomatic	Pb	7.6	None	NEN, SPN	MP	Panc	None	70 months, no recurrence^b^
5	Chetty et al. [5]	2009	67, F	Asymptomatic	Ph	2.6	None	NEN	PD	Panc	None	6 months, no recurrence
6	Sugawara et al. [6]	2010	55, F	Asymptomatic	Ph	7.0	None	N.A	PD	Panc	None	3 years, no recurrence^b^
7	Santos et al. [7]	2012	40, F	Asymptomatic	Pb	3.0	FNA (insufficient material)	N.A	Partial pancreatectomy	Panc	None	N.A
8	Tasdemir et al. [8]	2012	24, F	Abdominal pain, constipation	Ph	18.5	None	Mesenchymal tumor	EN	Panc	None	3 months, no recurrence
9	Azadi et al. [9]	2012	57, M	Asymptomatic	Pt	3.1	Biopsy (spindle cell neoplasm)	Spindle cell neoplasm	DP and left nephrectomy	Panc	None	N.A
10	van der Vorst et al. [10]	2012	67, F	Abdominal pain	Ph	2.8	None	NEN	EN	Panc	None	N.A
11	Patel et al. [11]	2013	43, M	Pancreatitis	Ph	6.0	EUS-FNA (SFT)	Metastatic SFT	PD and partial hepatectomy	Kidney	N.A	1.5 years, no recurrence
12	Osuga et al. [12]	2014	62, F	Asymptomatic^c^	Phb	3.4	EUS-FNA (SFT)	Metastatic SFT	MP	CNS	High mitotic rate	N.A
13	Estrella et al. [13]	2015	52, F	Jaundice, loss of appetite	Ph	15.0	None	NEN	PD	Panc	Hypercellularity, high mitotic rate, cytological atypia, necrosis	40 months, no recurrence
14	Baxter et al. [14]	2015	58, F	Abdominal pain	Ph	3.5	EUS-FNA (non-diagnostic)	NEN, GIST, sarcoma	PD	Panc	None	1 year, no recurrence
15	Paramythiotis et al. [15]	2016	55, M	Abdominal pain	Pb	3.6	None	NEN, SPN, GIST	SPDP	Panc	Necrosis	12 months, no recurrence
16	Murakami et al. [16]	2016	82, M	Edema, hypertension, hypokalemia	Pt	6.0	None	NEN	DP	Panc	N.A	4 months, died of sepsis
17	Spasevska et al. [17]	2016	47, M	Abdominal pain, jaundice	Ph	3.5	None	Cystadeno-carcinoma	PD	Panc	None	1 week, died of complications
18	Sheng et al. [18]	2017	1, M	Jaundice	Ph	2.0	None	N.A	PD	Panc	High mitotic rate, cytological atypia	12 months, no recurrence
19	Colvin et al. [19]	2017	76, M	Fatigue, abdominal pain, weight loss	Pbh	1.5	EUS-FNA (SFT)	Metastatic SFT	Robotic CP	Chest wall	Hypercellularity	1 year, recurrence in the chest wall
20	D’Amico et al. [20]	2017	52, M	Asymptomatic	Pt	2.0	None	NEN	EN	Panc	None	24 months, no recurrence
21	Oana et al. [21]	2017	73, M	Abdominal discomfort	Ph	6.5	None	NEN, ACC, GIST	Partial pancreatectomy	Panc	None	3 years, no recurrence
22	Current case	2019	58, F	Asymptomatic	Pt	5.6	None	NEN, SPN, MCN	DP	CNS	High mitotic rate	4 years, meningeal dissemination

*ACC* acinar cell carcinoma, *CNS* central nervous system, *CP* central pancreatectomy, *DP* distal pancreatectomy, *EN* enucleation, *EUS* endoscopic ultrasonography, *FNA* fine-needle aspiration, *GIST* gastrointestinal stromal tumor, *MCN* mucinous cystic neoplasm, *MP* middle pancreatectomy, *N.A* not available, *NEN* neuroendocrine neoplasm, *Panc* pancreas, *PD* pancreaticoduodenectomy, *SFT* solitary fibrous tumor, *SPDP* spleen-preserving distal pancreatectomy, *SPN* solid pseudopapillary neoplasm

^a^Malignant findings of SFT according to the World Health Organization classification [29]

^b^Information in the postoperative course was retrieved from the report by Baxter et al. [14]

^c^The patient had a chest discomfort, which was considered irrelevant to the pancreatic disease

With regard to the imaging findings, pancreatic SFTs show similar characteristics to those of tumors with pleural origin [6]. Contrast-enhanced CT and MRI show an enhancement through the arterial to portal phase, mimicking a tumor of neuroendocrine origin [5]. Consequently, the tumors were preoperatively diagnosed as neuroendocrine neoplasms in most of the previous reports [1, 2, 4, 5, 10, 13–16, 20, 21]. MRI generally demonstrates hypo- or isointensity on T1-weighted imaging and hyperintensity on T2-weighted imaging [9], as seen in the present case. In contrast, some cases showed different signal intensity because of the fibrotic components within the tumors [6, 12, 16, 18, 19]. According to previous reports, some pancreatic SFTs show heterogenous enhancement similar to the current case [6, 13, 18, 21], presumably depending on the volume of collagen within the tumors [18]. Furthermore, non-enhancing portions can be sometimes found, which mostly derived from cystic degeneration [4, 7, 11, 12, 15] and rarely from tumor necrosis [13] or retention cyst [17]. These findings are, however, not characteristic of SFTs and thus cannot be regarded as distinct imaging features.

SFT does not have any definitive clinical features or imaging findings, thus making preoperative diagnosis challenging. Generally, accurate diagnosis is made via pathological examination of a resected specimen. Image-guided pancreatic biopsy such as EUS-FNA or CT-guided biopsy might be useful for a precise preoperative diagnosis. However, although mesenchymal tumors may be diagnosed using these procedures [3, 9], preoperative differentiation of SFT from other spindle cell tumors of mesenchymal origin is yet to be achieved except for a limited number of cases of postoperative metastases [11, 12, 19]. Although the present case was a metastatic disease, an accurate diagnosis could not be obtained preoperatively because we refrained from aspiration biopsy to avoid bleeding and tumor dissemination based on imaging findings.

Histopathologically, SFT is composed of spindle-shaped cells growing in a patternless or hemangiopericytomatous arrangement and exhibiting a stroma with markedly varying amounts of collagen [1, 4]. Entrapped normal pancreatic acinar and ductal tissues are occasionally detected in the tumor [5]. However, these features do not necessarily differentiate SFT from other spindle cell tumors. Immunohistochemical analysis remains essential in establishing a definitive diagnosis of SFT. SFT typically stains positively for CD34, Bcl-2, and CD99, while it is generally negative for cytokeratin, epithelial membrane antigen, smooth muscle actin, desmin, S-100, c-kit, chromogranin, and synaptophysin. CD34, Bcl-2, and CD99 have been recognized as a representative marker of SFT, but they can also be positive in other neoplasms. Recent studies show that the *NAB2-STAT6* gene fusion can be detected in the majority of SFTs at any anatomic sites, and nuclear expression of STAT6 reflects the presence of *NAB2-STAT6* gene fusion. Thus, STAT6 expression in the nuclei is now considered highly specific for SFTs [30, 31]. In the present case, the tumor was sparsely positive for CD34, which was an untypical finding of SFT, but we established the diagnosis of SFT based primarily on diffuse nuclear expression of STAT6.

Although most SFTs are benign, 10–15% of SFTs localized in the extrapleural space are malignant and recur [8]. The histologic criteria for malignant SFTs are yet to be established, but the World Health Organization (WHO) states that the presence of hypercellularity, increased mitotic rate (> 4 mitoses per 10 high-power fields), cytological atypia, tumor necrosis, and infiltrative margins suggest malignancy [29]. In addition, Yokoi et al. reported that marked diminution or complete loss of CD34-positive cells is a characteristic of malignant transformation of SFTs [32]. Further, positive surgical margins and a tumor size greater than 10 cm predicted worse clinical outcome [33]. In the current case, the tumor was not only a metastatic disease but also histopathologically exhibited a high mitotic rate (10 mitoses per 10 high-power fields) and sparse staining for CD34, indicating its malignant potential.

The present case is remarkable in that the pancreatic lesion was a metastasis primarily derived from intracranial SFT and that the correct diagnosis could not be made until the metastatic lesion of the pancreas was resected and histopathologically examined. SFTs in the central nervous system had not been previously recognized until the first case series of SFTs involving the meninges was reported in 1996 [34]. Because of its rarity and resemblance to other common tumors, intracranial SFT can occasionally be misdiagnosed as a meningioma. The patient underwent the first operation for the brain tumor in 1983, which caused the misdiagnosis of the tumor. However, according to the latest WHO classification [35], solitary fibrous tumors can be readily differentiated immunohistochemically from meningioma based on its STAT6 nuclear expression, with the latter being negative for STAT6.

Hemangiopericytoma (HPC), which was previously classified as a distinct tumor entity, is now considered identical to SFT based on its immunostaining profile. HPCs behave more aggressively and consequently have a higher risk of postoperative recurrence. If the first operation had been performed with adequate immunohistochemical analysis, a precise diagnosis of malignant SFT, i.e., HPC, might have been obtained much earlier considering its clinicopathological features.

Complete resection with clear surgical margins is the optimal treatment for SFT, and it yields good outcomes [33]. With regard to local recurrence, 5–7% of patients who underwent surgical resection of SFTs developed local relapse [33, 36]. However, the correlation between margin status and local recurrence remains to be completely examined and is controversial. Moreover, favorable outcomes have been reported in patients treated with enucleation [2, 8, 20]. Thus, further investigations are needed to establish the standard surgical procedure for SFTs, including the optimal resection margin. The presence of a malignant component in SFT has been associated with postoperative recurrence and metastasis and tumor-related death [13], and thus, close long-term follow-up is required even after a complete resection in tumors showing malignant behavior.

## Conclusions

SFT should be considered in the differential diagnosis of untypical hypervascular pancreatic mass, particularly in patients with a history of intrathoracic or intracranial mesenchymal tumor. Currently, accurate diagnosis of SFT relies on immunohistochemical analysis of the resected specimen, but recent progress in immunohistochemistry may lead to the preoperative diagnosis of this tumor entity. Hyperproliferative status indicates a malignant disease and requires attentive postoperative follow-up.

## Data Availability

Data sharing is not applicable to this article as no datasets were generated or analyzed during the current study.

## Abbreviations

CT: Computed tomography
EUS: Endoscopic ultrasonography
EUS-FNA: EUS-guided fine-needle aspiration biopsy
HPC: Hemangiopericytoma
MRI: Magnetic resonance imaging
SFT: Solitary fibrous tumor
WHO: World Health Organization
